# Homœopathy

**Published:** 1857-02

**Authors:** Robert E. Campbell

**Affiliations:** Benton, Lowndes County, Alabama


### ARTICLE III.

*Homœopathy.* By ROBERT E. CAMPBELL, M. D., Benton, Lowndes County, Alabama.

[CONTINUED FROM PAGE 198.]

The dogma of "*similia similibus curanter*" has received some farcical applications, and introduced into the homœopathic pharmacy, substances which can only be tolerated in ordinary stomachs in the 6,000th dilution. It is announced, with "a feeling of inward satisfaction," by Dr. (?) Muir, that he has rendered "a real service to the theory and practice of medicine;" for, by deep research into the mysterious laws of Nature, he has unlocked one of the "wonderful coincidences"

of the aforesaid *Dame*, and discovered a most valuable specific for various infantile diseases—most especially for *itch.*

The aforesaid Dr. M. made a very extraordinary observation, that the heads of children were frequently the geographical seat of insectile occupation, which was attended by a peculiar sensation very similar to that experienced about the knuckles when colonized by a race of subcutaneous inhabitants. These startling facts set the brain of the *Dr.* to itching. Surely, thought he, these curious creatures were not made in vain; hence they were made for something. Now, as to what that *something* was; and the fertile brain of the sagacious *Dr.* was not long at a loss; for, he argued that they were indigenous to children, and therefore, they must be good for children! Sublime theory! Newton, in his palmiest days, has been distanced by a modern Dr. (?) But to return. The Dr. went on tracing the striking analogy: he argued that they were products of the body of the child, yet they did no good *outside*, and, as a matter of course, they must do some *within.* Without more ado, one of these quiet colonies was invaded, suitable specimens captured by the *Dr.*, (who seems to have been a connoisseur,) a "*tea*" was concocted, and he began his experiments. Symptoms were observed in the *stomach* (*nausea?*), head (*crawling?*), skin (*itching?*), etc.; and according to *Dr.* Simpson, he devotes twelve pages to a description of these *startling* symptoms. Twelve pages to record the infinitesimal powers of lice. But, Dr. Muir is not the only genius who has enlivened the pages of the *materia medica* by his researches into animated nature.

One of the *followers*, I know not who, startled the *infinitesimal world*, by the announcement, that the itch insect, triturated with sugar, had been found eminently useful for "tingling digits;" and those horrible pests to nude and weary humanity, yclept "*bugs*," when sufficiently sweetened or dissolved, (for which purpose infinite sugar and universal water hardly seem adequate,) are declared to be "a sovereign balm for their own venomous stings." We must confess there is a grim satisfaction in thus turning the tables upon the vermin. There is a vindictive pleasure in seeing "the biter bit," and we must all allow the infinitesimals to wage a war of extermination upon "bugs." And every homœopathic Dr. should seize upon all occasions to fill his bottle: then his visits to the nursery would not be without some good result.

The homœopathic publications publish many other substances equally disgusting, but we will spare our readers the nauseous information; yet, at the same time, we would beg our fastidious friends of both sexes, not to ask any questions of their homœopathic physicians as to the contents of his infinite pills; for

"Where ignorance is bliss, 'tis folly to be wise."

We have heretofore noticed the conceit of Hahnemann, that most, if not all, the diseases to which frail humanity are liable, are attributable to the obscure constitutional workings of the *psora* or itch insect. It is and will be a mystery, deep and unfathomable, how the great philosopher arrived at this conclusion—by what process of reasoning he satisfied himself (?) of the plausibility of his startling theory. Hahnemann, in his "*Organon*," says he "spent twelve years in finding out this thousand headed monster of disease," which he finally found out to be the cause of "nervous debility, hysteria, hypochondriasis, mania, melancholia, imbecility, madness, epilepsy, convulsions, mollities ossium, cancer, gout, jaundice, dropsy, hemorrhages, deafness, blindness, palsy," etc., etc. How grateful should we be to Hahnemann for thus finding a specific for most human maladies; and how fortunate that so benevolent a provision for mankind is found in the heads of children! And how sad to think of the wanton destruction, nay, butchering, of the useful insect by unreflecting mothers! Down with the ignorant and shameful device of combs; lay an injunction on thumb and finger; send every suspected head to the Doctor to be looked at, and let poor afflicted man have dealt out to him his allotted portion of "tea!"

A thousand striking "coincidences," never before understood, now begin to unfold themselves, showing the obscure workings of a grand truth before unperceived! Has not man always expected suggestions to come from the head? Does he not, when in perplexity, scratch his scalp? In mania, does he not tear his hair? In melancholia, etc., does not a blister there imitate *pediculoid* irritation? Is not a doctor a *lice*-entiate? Is not the extreme of calamity expressed, by "as dead as a nit?" What will be thought of these "coincidences" we are unable to say, yet, if there is not a deep and mysterious meaning in them, we know not what is to become of the law of "*similia similibus*," for no homœopathic resemblances are so striking as those above recorded.

But homœopathy has shed its light on moral and religious, as well as on physical and animated things; and a sort of phosphorescent gleam it is. The first great truth discerned by it was, that Hahnemann was inspired, and Homœopathy a revelation; that "the new evangelist" was sent "to render medicine, like other sciences, properly Christian!" And we are gravely informed, that he has faithfully executed his commission, and "through him Christian science became universal, and redemption descended from the dominion of sentiment to that of the ideas of intelligence."

We confess the above quoted paragraph is very obscure; we cannot get the clue to it, unless is meant by "Christian science" the art of catching and tea-drawing the universal remedy; and "redemption descending from the dominion of sentiment, to that of ideas and intelligence," may mean the transfer of the happy conception of Dr. Muir, to actual manipulations upon the seat of intelligence of one of his children. Dr. Muir, who is himself pronounced to be an "apostle of homœopathy," says, that "homœopathy is not a science, merely, but also for those who comprehend it, a sublime devotion; a form of religion; a divine rainbow of union, holding out to mankind the promise of speedy regeneration." We must spare our readers his argument in favor of the divinity of the system, from the idea of the infinity suggested by its doses. The crack-brained man actually perceives no difference between infinite emptiness and infinite fullness. But enough of Dr. Muir; we have used him *ad nauseam usque.*

The attempt to connect homœopathy with religion, and to promulgate a system of blasphemous infidelity, under the name of a newly developed Christianity, is by no means confined to a few crazy transcendentalists, who can manage to write in appropriate German and French. The *English* homœopathic journals furnish us with melancholy evidences of the spread of the infection in the land of bibles. Professor Simpson has furnished us with several quotations upon the subject in his valuable work against the homœopaths, to which we must refer our readers. We have only to add, that homœopathic religion is worthy of homœopathic physic—equally true, reasonable and effective.

We now await the next folly; can it equal the last?

